# Autoantibodies to a 140-kd protein in juvenile dermatomyositis are associated with calcinosis

**DOI:** 10.1002/art.24547

**Published:** 2009-06

**Authors:** H Gunawardena, L R Wedderburn, H Chinoy, Z E Betteridge, J North, W E R Ollier, R G Cooper, C V Oddis, A V Ramanan, J E Davidson, N J McHugh, Juvenile Dermatomyositis Research Group, UK and Ireland

**Affiliations:** 1 Royal National Hospital for Rheumatic Diseases, and University of BathBath, UK; 2 Institute of Child Health, University College LondonLondon, UK; 3 Salford Royal NHS Foundation Trust, and University of ManchesterManchester, UK; 4 University of BathBath, UK; 5 University of ManchesterManchester, UK; 6 University of PittsburghPittsburgh, Pennsylvania; 7 Royal National Hospital for Rheumatic Diseases, Bath, and Bristol Royal Hospital for ChildrenBristol, UK; 8 Royal Hospital for Sick ChildrenGlasgow, UK

## Abstract

**Objective:**

The identification of novel autoantibodies in juvenile dermatomyositis (DM) may have etiologic and clinical implications. The aim of this study was to describe autoantibodies to a 140-kd protein in children recruited to the Juvenile DM National Registry and Repository for UK and Ireland.

**Methods:**

Clinical data and sera were collected from children with juvenile myositis. Sera that recognized a 140-kd protein by immunoprecipitation were identified. The identity of the p140 autoantigen was investigated by immunoprecipitation/immunodepletion, using commercial monoclonal antibodies to NXP-2, reference anti-p140, and anti-p155/140, the other autoantibody recently described in juvenile DM. DNA samples from 100 Caucasian children with myositis were genotyped for HLA class II haplotype associations and compared with those from 864 randomly selected UK Caucasian control subjects.

**Results:**

Sera from 37 (23%) of 162 patients with juvenile myositis were positive for anti-p140 autoantibodies, which were detected exclusively in patients with juvenile DM and not in patients with juvenile DM–overlap syndrome or control subjects. No anti-p140 antibody–positive patients were positive for other recognized autoantibodies. Immunodepletion suggested that the identity of p140 was consistent with NXP-2 (the previously identified MJ autoantigen). In children with anti-p140 antibodies, the association with calcinosis was significant compared with the rest of the cohort (corrected *P* < 0.005, odds ratio 7.0, 95% confidence interval 3.0–16.1). The clinical features of patients with anti-p140 autoantibodies were different from those of children with anti-p155/140 autoantibodies. The presence of HLA–DRB1*08 was a possible risk factor for anti-p140 autoantibody positivity.

**Conclusion:**

This study has established that anti-p140 autoantibodies represent a major autoantibody subset in juvenile DM. This specificity may identify a further immunogenetic and clinical phenotype within the juvenile myositis spectrum that includes an association with calcinosis.

Juvenile dermatomyositis (DM) is the most common of the idiopathic inflammatory myopathies (IIMs) of children. The reported incidence is 0.8–4.1 per million children per year (1–3). Juvenile DM is chronic, potentially debilitating, and can be associated with significant morbidity. Due to the heterogeneity of the condition with multisystem disease, the clinical outcome (and thus prognosis) is difficult to predict. Certain clinical features, such as skin ulceration, calcinosis, gastrointestinal involvement, and respiratory disease, have been proposed as predictors of a severe disease course in juvenile DM (4–7).

The precise etiology of IIMs is unknown, but there is increasing evidence to suggest an important role for autoimmunity. Knowledge of an autoantibody profile is an important cornerstone in the diagnosis of patients with a wide variety of autoimmune connective tissue disorders. Myositis-specific autoantibodies (MSAs) are being observed with increasing frequency in adult patients with IIM. There is now increasing evidence that MSAs are associated with homogeneous clinical subsets within the IIM spectrum, which can help predict clinical outcomes (8–10). For example, autoantibodies directed against the aminoacyl–transfer RNA synthetases (aaRS) form the largest group of MSAs in adult IIM and are associated with the antisynthetase syndrome (10,11). Other well-described MSAs in adult IIM that are associated with specific clinical manifestations include anti-signal recognition particle (anti-SRP) and anti–Mi-2 autoantibodies (10).

To date, MSAs in juvenile myositis, including juvenile DM, have been less well characterized. Previous reports have described myositis-associated autoantibodies (MAAs), including anti–PM-Scl and anti–U1 RNP, in juvenile DM overlap syndromes (12). Anti–Mi-2 has been described more frequently, but this autoantibody specificity and others such as aaRS and anti-SRP are detected in a relatively small number of juvenile myositis cases (13–15). Recently, our group and other investigators have observed that autoantibodies to a 155-kd protein and a 155/140-kd doublet protein are a major serologic subset in juvenile DM (16,17). In addition, anti-p155/140 autoantibodies appear to define a distinct clinical phenotype within the juvenile DM spectrum (17). A further autoantibody termed anti-MJ, which targets a 140-kd protein, has been described in a US cohort of patients with juvenile DM (18). The MJ autoantigen was recently identified as nuclear matrix protein NXP-2 (19).

In this study, we describe the prevalence, clinical associations, and immunogenetic associations of autoantibodies targeting a p140 protein in children recruited to the Juvenile DM Registry and Repository for UK and Ireland (JDRR) (for review, see refs.^6^ and^12^). We demonstrate that anti-p140 and anti-p155/140 are different autoantibody subsets and investigate the identity of the p140 target, which is likely to be the same as the previously identified MJ autoantigen NXP-2 (also termed MORC3) (18,19).

## PATIENTS AND METHODS

### Patients and sera

The JDRR has recruited patients with juvenile-onset myositis, all of whom were younger than age 16 years at the time of disease onset and diagnosis, from 10 centers around the UK (6). All patients had probable or definite myositis according to the diagnostic criteria described by Bohan and Peter (20,21). Demographic and serial clinical data were recorded at the time of diagnosis and prospectively at subsequent visits (approximately every 6 months). The clinical information recorded comprised specific cutaneous manifestations, including the presence of Gottron's lesions, skin ulceration, edema, calcinosis, and the distribution of skin rash over the body. Details on muscle involvement included muscle enzymes (creatine kinase [CK] and lactate dehydrogenase [LDH]) and the Childhood Myositis Assessment Scale (CMAS) (22) at baseline. Data were stored onto a central database, using anonymous codes. Serum and DNA samples were obtained at the time of diagnosis and stored at −20°C until required. Serum samples for serologic typing were available from 162 children recruited to the registry. DNA for genotyping was available from 100 Caucasian children with juvenile DM.

Clinical data were available for 160 children, 74% of whom were female. The median age at disease onset was 6 years (interquartile range [IQR] 3–9 years), and the median age at the time of diagnosis was 7 years (IQR 4–10 years). For this study, the median followup period from disease onset to the time of data analysis was 48 months (IQR 33–72 months) for the overall cohort. One hundred thirty-seven children had juvenile DM. Juvenile DM–scleroderma (juvenile DM–SSc) overlap syndrome is well recognized in children with juvenile DM who have a history of Raynaud's phenomenon, sclerodactyly, and other sclerodermatous skin changes. In this study, 21 children were defined as having juvenile DM–SSc overlap syndrome, with 2 or more of the above-mentioned features. Two children were defined as having other forms of juvenile myositis, not specifically juvenile DM or juvenile DM–SSc overlap syndrome.

Sera from 124 juvenile disease control subjects (20 with scleroderma/linear scleroderma, 8 with systemic lupus erythematosus, and 96 with juvenile idiopathic arthritis) were also analyzed. No sera from healthy children were available, reflecting the ethical difficulties in studies of this nature. Therefore, sera from 50 healthy adult control subjects were also serotyped.

### Ethics approval

All patients or their parents gave fully informed written/parental consent to participate and provide biologic samples, according to the Declaration of Helsinki, under both national multicenter and local ethics committee regulations.

### Indirect immunofluorescence

Indirect immunofluorescence was performed by standard methods, using Hep-2 cells and fluorescein-labeled anti-human IgG (Sigma, Poole, UK).

### Protein immunoprecipitation (IP)

IP with K562 cell extracts was performed as previously described (23). Briefly, 10 μl of sera was mixed with 2 mg protein A–Sepharose beads (Sigma) in IP buffer (10 m*M* Tris HCl, pH 8.0, 500 m*M* NaCl, 0.1% volume/volume Igepal) at room temperature for 30 minutes. Beads were washed in IP buffer prior to the addition of 120 μl ^35^S-methionine–labeled K562 cell extract. Samples were mixed at 4°C for 2 hours. Beads were washed in IP buffer followed by Tris buffered saline buffer (10 m*M* Tris HCl, pH 7.4, 150 m*M* NaCl) before being resuspended in 50 μl sodium dodecyl sulfate (SDS) sample buffer (Sigma). After heating, proteins were fractionated by 10% SDS–polyacrylamide gel electrophoresis, enhanced, fixed, and dried. Labeled proteins were analyzed by autoradiography.

### IP with mouse monoclonal anti–NXP-2 and immunodepletion experiments

Ten microliters of anti-p140–positive sera or 50 μl of commercial mouse antibody to NXP-2 (MORC3) (Medical and Biological Laboratories, Nagoya, Japan) was mixed with 100 μl of prewashed protein G Dynabeads (Dynal, Liverpool, UK) in sodium phosphate (pH 8.1, 0.1*M*) at room temperature for 30 minutes. The antigens were immunoprecipitated as described for IP, using ^35^S-methionine–labeled K562 cell extracts. Immunodepletion was performed to ascertain whether the IP pattern observed with anti-p140–positive juvenile DM sera and the commercial antibody to NXP-2 was attributable to precipitation of the same antigen. This method was also used to confirm that anti-p155/140–positive juvenile DM sera target a different protein (17). Cell extracts were depleted of autoantibody targets, using a reference anti-p140–positive juvenile DM serum, reference anti-p155/140–positive juvenile DM serum, and normal serum as a negative control.

Briefly, duplicate samples, each of which contained 10 mg protein A–Sepharose beads (when preparing predepleted p140 cell extract for IP with commercial anti–NXP-2, 150 μl prewashed protein G Dynabeads was used) in 1 ml IP buffer and 50 μl anti-p140 serum (or 50 μl anti-p155/140 serum) were mixed at room temperature for 30 minutes. The beads were washed in IP buffer, and 1 tube (tube A) was placed on ice, while 120 μl ^35^S-methionine–labeled K562 cell extract and 380 μl IP buffer were added to tube B. Tube B was mixed at 4°C for 2 hours; the supernatant was transferred to the corresponding tube A, which was then mixed at 4°C for an additional 2 hours. The supernatant from the corresponding tube A (i.e., p140 antigen–depleted cell extract or p155/140 antigen–depleted cell extract) was stored at −80°C. IP with depleted cell extracts was completed using 50 μl commercial anti–NXP-2, different anti-p140 sera (10 μl), or different anti-p155/140 sera (10 μl).

### HLA typing

DNA samples from Caucasian patients with juvenile DM were extracted from blood samples and analyzed using a standard phenol–chloroform method. Patients were broad-typed for the HLA–DRB1 and DQB1 loci using a commercially available polymerase chain reaction sequence-specific oligonucleotide probe typing system (Dynal, Hamburg, Germany), as previously described (24). The HLA–DQA1 data were derived from the DRB1 and DQB1 results, using well-documented Caucasian haplotype tables (25).

### Statistical analysis

Clinical and HLA allelic associations were derived from 2 × 2 contingency tables, using the chi-square test or 2-tailed Fisher's exact test when the value for individual cells was ≤5. Significant results were expressed as odds ratios (ORs) with exact 95% confidence intervals (95% CIs). *P* values were adjusted using the Bonferroni correction when comparing clinical associations. Uncorrected *P* values are presented for possible immunogenetic associations. Haplotypes were estimated for selected loci using the expectation-maximization algorithm, as implemented in HelixTree (version 3.1.2; Golden Helix, Bozeman, MT) (22). SPSS for Windows (version 14; SPSS, Chicago, IL) (for clinical data) and Stata (release 9; Stata Corp., College Station, TX) (for HLA data) were used to perform statistical analysis.

## RESULTS

### Identification of anti-p140 autoantibodies in juvenile DM sera

**Figure 1:**
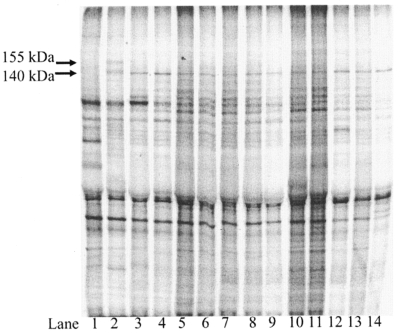
Immunoprecipitation of p140 autoantigens. The autoradiogram shows the results of 10% sodium dodecyl sulfate–polyacrylamide gel electrophoresis of immunoprecipitates of ^35^S-methionine–labeled K562 cell extracts, using normal serum (lane 1), anti-p155/140–positive juvenile dermatomyositis (DM) serum (lane 2), and different anti-p140–positive juvenile DM sera (lanes 3–14).

Following IP, sera from several patients with juvenile DM recognized a distinct protein band with a molecular weight of ∼140 kd (Figure 1). No anti-p140–positive sera were observed to immunoprecipitate any other known MSAs or MAAs. A weak nonspecific nuclear pattern (or, in some cases, antinuclear antibody negativity) was observed by indirect immunofluorescence in all anti-p140 sera (data not shown). Anti-p140 was not detected in any of the juvenile disease control sera or healthy adult control sera.

### Confirmation of the p140 autoantigen

**Figure 2:**
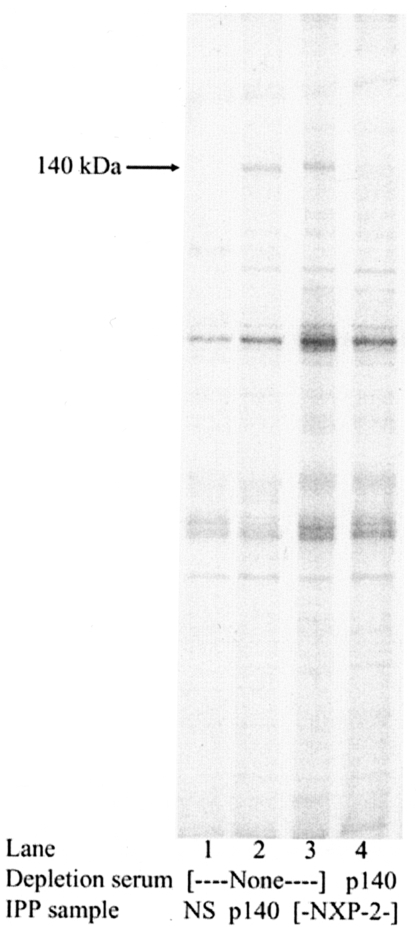
Immunodepletion of commercial NXP-2. The autoradiogram shows the results of 10% sodium dodecyl sulfate–polyacrylamide gel electrophoresis of immunoprecipitates of ^35^S-labeled K562 cell extracts using normal sera (NS; lane 1), reference anti-p140–positive juvenile dermatomyositis (DM) serum (lane 2), and commercial anti–NXP-2 (lane 3). Lane 4 shows the results of immunoprecipitation using commercial anti–NXP-2 with ^35^S-methionine–labeled cell extract predepleted with reference anti-p140–positive juvenile DM serum. IPP = immunoprecipitation.

**Figure 3:**
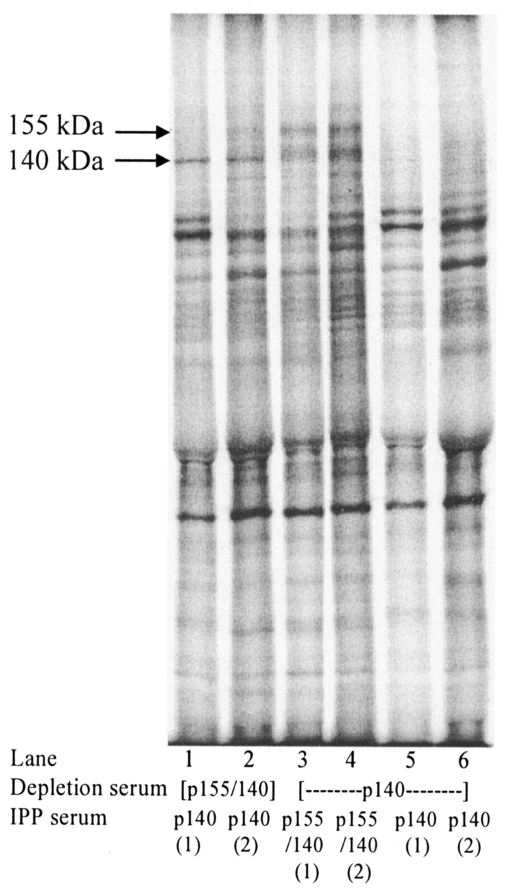
Immunodepletion experiments with anti-p140 and anti-p155/140. The autoradiogram shows the results of 10% sodium dodecyl sulfate–polyacrylamide gel electrophoresis of immunoprecipitates, using different anti-p140–positive juvenile DM sera (lanes 1, 2, 5, and 6) and anti–p155/140–positive juvenile DM sera (lanes 3 and 4). Immunoprecipitation was performed with ^35^S-methionine–labeled K562 cell extracts predepleted with either reference anti-p140–positive juvenile DM serum or reference anti-p155/140–positive juvenile DM serum. See Figure 2 for definitions.

Our results suggest that the p140 protein targeted by juvenile DM sera in our study has the same identity as the MJ antigen, NXP-2 (19). Using a commercial antibody raised against NXP-2, IP resulted in the precipitation of a band with the same molecular weight and IP pattern as those observed in anti-p140–positive juvenile DM sera (Figure 2, lane 3). When the cell extract was predepleted with juvenile DM anti-p140–positive sera, the IP band present with commercial anti–NXP-2 was no longer detectable (Figure 2, lane 4). The immunodepletion results support the co-identity of the p140 protein precipitated by different anti-p140–positive juvenile sera (complete data not shown). Figure 3 (lanes 5 and 6) shows an example in which the 140-kd band is no longer detectable following IP with anti-p140 sera (designated “1”) and using predepleted reference p140 cell extract (designated “2”). In addition, immunodepletion confirmed that anti-p140 sera target a different protein to juvenile DM sera that recognizes the p155/140 doublet protein (Figure 3, lanes 1–4, showing that the respective bands are still present following IP).

### Clinical associations of anti-p140 autoantibodies

Of 162 juvenile myositis sera serotyped by IP, 37 (23%) had anti-p140 autoantibodies. This autoantibody specificity was observed exclusively in sera from patients with juvenile DM, with a frequency of 27%. Anti-p140 was not detected in any sera from patients with juvenile DM–SSc overlap syndrome. Selected clinical features of anti-p140–positive patients compared with the overall anti-p140–negative juvenile myositis cohort are shown in Table 1. There was no significant difference in the female-to-male ratio, the age at the time of disease onset or diagnosis, and disease duration (from disease onset to the time of this study) between anti-p140–positive and anti-p140–negative patients. Overall, there was no significant difference in the presence of Gottron's lesions, skin ulceration, edema, or the distribution of rash, except anti-p140–positive children had no rashes on the trunk compared with anti-p140–negative children (corrected *P* [*P*_corr_] = 0.02). Anti-p140–positive cases were significantly associated with the presence of subcutaneous calcinosis compared with anti-p140–negative cases (*P*_corr_ < 0.005, OR 7.0, 95% CI 3.0–16.1). There was no significant difference between other clinical features when comparing anti-p140–positive patients and anti-p140–negative patients, including the baseline CK level, the CMAS score, the Childhood Health Assessment Questionnaire (C-HAQ) score (26), the physician's global assessment scale (PGA) score, and the presence of arthritis, Raynaud's phenomenon, dysphagia, mouth ulcers, and alopecia (data not shown).

**Table 1:** Selected clinical features of anti-p140–positive patients with juvenile DM compared with anti-p140–negative juvenile DM patients with myositis and anti-p155/140–positive juvenile DM patients*

Characteristic	Anti-p140 positive (n = 37)†	Anti-p140 negative (n = 125)†	Anti-p155/140 positive (n = 28)
Age at disease onset, median (IQR) years	6 (2–10)	6 (4–9)	6 (4–10)
Age at diagnosis, median (IQR) years	7 (4.5–10.3)	7 (4–10)	7 (4–10)
Female sex	72	74	57
Type of skin lesion			
Gottron's papules	85	82	100
Ulceration	34	23	57
Edema	35	35	64
Calcinosis	54‡	15	14
Lipoatrophy	18	13	18
Distribution of skin rash			
Periorbital	79	69	96
Periungal	64	67	86
Small joints	67§	72	96
Large joints	52	51	75
Trunk	0¶	18	32

* Except where indicated otherwise, values are the percent of patients. DM = dermatomyositis; IQR = interquartile range.

† Not all patients had clinical data available for each feature.

‡ Corrected *P* (*P*_corr_) < 0.005, odds ratio (OR) 7.0, 95% confidence interval (95% CI) 3.0–16.1 versus anti-p140–negative patients, and *P*_corr_ = 0.015, OR 7.1, 95% CI 2–25 versus anti-p155/140–positive patients.

§ *P*_corr_ = 0.05, OR 13.5, 95% CI 2–113 versus anti-p155/140–positive patients.

¶ *P*_corr_ = 0.02 versus anti-p140–negative patients, and *P*_corr_ < 0.005 versus anti-p155/140–positive patients.

Possible clinical differences were noted when anti-p140–positive patients were compared with anti-p155/140–positive patients (Table 1). Overall, the age at disease onset or diagnosis and disease duration (for anti-p140–positive patients, median 48 months [IQR 34–72]; for anti-p155/140–positive patients, median 52 months [IQR 36–84]) were similar between the 2 autoantibody groups. Compared with anti-p155/140–positive patients, anti-p140–positive patients had an association with calcinosis (14% versus 54%; *P*_corr_ = 0.015, OR 7.1 [95% CI 2–25]). In contrast, anti-p155/140–positive patients compared with the anti-p140–positive group had a higher frequency of ulceration and cutaneous edema; however, this result was not significant after correcting for multiple comparisons. The distribution of rash was wider on the trunk (*P*_corr_ < 0.005) and over the small joints (*P*_corr_ = 0.05, OR 13.5 [95% CI 2–113]) in anti-p155/140–positive children compared with anti-p140–positive patients.

At the time of diagnosis, anti-p140–positive compared with anti-p155/140–positive children had a nonsignificantly lower CK level (median 202 IU/liter [IQR 76–2,142] versus median 571 IU/liter [IQR 234–2,495]) and LDH level (median 845 IU/liter [IQR 710–1,620] versus median 1,171 IU/liter [IQR 736–1,647]). In addition, anti-p140–positive patients had a higher CMAS score at the time of diagnosis compared with anti-p155/140–positive patients (median 42 [IQR 23–49] versus median 16 [IQR 7–38]; *P* not significant after adjustment for multiple comparisons). There was a nonsignificant trend toward a lower baseline C-HAQ score and PGA score in the anti-p140 group compared with the anti-p155/140 group (median C-HAQ score 1.31 [IQR 0.75–1.63] versus median 1.63 [IQR 0.78–2.34]); median PGA score 5 [IQR 3–7.4] versus median 7.3 [IQR 5–7.8]). However, these results need to be interpreted in the context that these data were recorded for only a small number of patients at the time of diagnosis.

### Immunogenetic associations of anti-p140 autoantibodies

HLA–DRB1*08 was a possible immunogenetic risk factor and was present in 23.5% of 17 anti-p140–positive patients compared with 5.4% of 864 control subjects (*P* = 0.01, OR 5.3 [95% CI 1.2–18.1]). HLA–DRB1*08 was observed in 4.8% of 83 anti-p140–negative patients and in 11.8% of 18 anti-p155/140–positive patients. DQA1*06 was also observed more frequently in anti-p140–positive patients (11.8% of 17 patients compared with 1% of 192 control subjects; *P* = 0.03, OR 12.7 [95% CI 0.8–181.0]). This haplotype was not observed in anti-p140–negative or anti-p155/140–positive patients. However, these results were not significant after correcting for multiple comparisons.

## DISCUSSION

There is now increasing evidence that MSAs are associated with (and thus can identify) clinical subsets within the adult IIM spectrum. Our study suggests that specific genetic and serologic profiles may also be associated with clinical phenotypes in juvenile DM (12–17). In this report, we describe the novel autoantibody specificity, anti-p140, in a UK cohort study of patients with juvenile DM. The presence of anti-p140 autoantibodies represents a major serologic subset in juvenile myositis; it was observed exclusively in juvenile DM and was not detected in any cases of juvenile DM–SSc overlap syndrome. All anti-p140–positive sera recognized the same polypeptide and did not immunoprecipitate any other known myositis autoantigens, including the anti-p155/140 autoantibody (16,17). Combining the data from our UK juvenile DM cohort studies, anti-p140 and anti-p155/140 autoantibodies were detected in ∼40% of patients with juvenile DM, in contrast to a much lower frequency of anti–Mi-2 and other myositis-specific or associated autoantibodies, as previously reported (12–15).

The confirmation of a further serologic subset in juvenile DM appears to have important clinical implications. Anti-p140 positivity was significantly associated with the presence of calcinosis when compared with the overall juvenile myositis cohort. In addition, anti-p140 and anti-p155/140 appear to define juvenile DM into 2 serologic subsets with more homogeneous clinical features. The results of this study, combined with our previous work (17), suggest that anti-p155/140–positive patients have a wider distribution of skin disease, more cutaneous complications including edema or ulceration, and possibly overall higher disease activity but a significantly lower frequency of calcinosis compared with anti-p140–positive patients.

The clinical differences observed between anti-p140–positive patients, anti-p155/140–positive patients, and patients with neither of these specificities are not explained by differences in the time to disease onset or disease duration. This is an interesting observation, because a factor suggested to influence the development of calcinosis is persistent active disease, including chronic cutaneous inflammation (27–29). The association between serotype and clinical phenotype suggests that the targeted autoantigens p140 and p155/140 may play a role in the pathogenesis of skin and soft tissue complications in juvenile DM. In addition, HLA–DRB1*08 is a possible immunogenetic risk factor for the development of anti-p140 autoantibodies in Caucasian children with juvenile DM.

Based on previous work that showed an association with the tumor necrosis factor α (TNFα) 308A allele, increased production of TNFα, and calcinosis (28), future studies to investigate for other potential susceptibility genes, including TNF polymorphisms, in juvenile DM patients with anti-p140 or anti-p155/140 autoantibodies would be of major interest.

The p140 protein targeted by an autoimmune response in our juvenile DM cohort study is consistent with NXP-2, the MJ autoantigen described in preliminary reports in a US juvenile DM cohort (18,19). NXP-2 has nuclear matrix–binding, RNA-binding, and coiled-coil domains that are structurally separated, which may implicate a role in diverse nuclear functions including regulation of transcriptional and RNA metabolism (30). Previously described DM-specific autoantigen targets in children and adults, Mi-2 and p155/140 (p155; transcription intermediary factor 1 gamma) (31,32), are nuclear proteins that also mediate gene transcription. It is of further interest to note that autoantibodies to small ubiquitin-like modifier activating enzyme (SAE), which is involved in posttranscriptional modification termed sumoylation, have been described in adult DM (33). To date, anti-SAE autoantibodies have not been detected in juvenile myositis sera (Gunawardena H: unpublished observations). However, NXP-2 has been shown to be a sumoylation target involved in transcriptional repression (34), which may suggest potential shared pathogenic mechanisms in both juvenile and adult DM.

In conclusion, anti-p140 autoantibody is a clinically important serologic marker that is observed with a high frequency in patients with juvenile DM and is associated with calcinosis, a complication of the disease that confers significant morbidity. In the future, routine testing for these novel autoantibodies (anti-p140 and anti-p155/140) at the time of disease onset could have prognostic value and identify those children who are at risk of more severe disease, which may influence management. Furthermore, increasing our understanding of autoimmune targets and their relationship to the clinical phenotype in juvenile myositis may provide further insight into pathogenic pathways that in turn will stimulate new therapeutic approaches.

## AUTHOR CONTRIBUTIONS

All authors were involved in drafting the article or revising it critically for important intellectual content, and all authors approved the final version to be published. Prof. McHugh had full access to all of the data in the study and takes responsibility for the integrity of the data and the accuracy of the data analysis.

**Study conception and design.** Gunawardena, Wedderburn, Chinoy, Betteridge, North, Cooper, Davidson, McHugh.

**Acquisition of data.** Gunawardena, Wedderburn, Chinoy, Betteridge, North, Cooper, Davidson, McHugh.

**Analysis and interpretation of data.** Gunawardena, Wedderburn, Chinoy, Betteridge, North, Ollier, Cooper, Oddis, Ramanan, Davidson, McHugh.

**Supervision of sample and data collection.** Wedderburn.
